# Data-Driven Parameter Identification of Synchronous Generators: A Three-Stage Framework with State Consistency and Grid Decoupling

**DOI:** 10.3390/s26072024

**Published:** 2026-03-24

**Authors:** Rasool Peykarporsan, Tharuka Govinda Waduge, Tek Tjing Lie, Martin Stommel

**Affiliations:** Department of Electrical and Electronic Engineering, Auckland University of Technology, Auckland 1010, New Zealand; rasool.peykarporsan@autuni.ac.nz (R.P.); tek.lie@aut.ac.nz (T.T.L.); mstommel@aut.ac.nz (M.S.)

**Keywords:** port-Hamiltonian systems, parameter identification, data-driven identification, physics-based regularization, energy-consistent modeling, Lyapunov stability, synchronous generator modeling, power system dynamics

## Abstract

As modern power systems grow increasingly complex, there is a pressing need for stability analysis methods capable of handling nonlinear dynamics while providing physically meaningful and reliable stability indices. Port-Hamiltonian (PH) frameworks have emerged as strong candidates in this regard, offering inherently stable formulations, energy-consistent representations, and modular plug-and-play scalability. However, the practical deployment of PH-based stability analysis remains hindered by the absence of reliable, high-fidelity parameter identification methods that rely on sensor measurements to capture system dynamics while remaining compatible with PH model structures. This paper addresses that gap by proposing a comprehensive three-stage data-driven identification framework for PH modeling of synchronous generators—the central dynamic component of any power system. While the IEEE Standard 115 provides established procedures for transient parameter identification, it exhibits fundamental limitations when applied to PH modeling, including single-scenario identifiability constraints, noise-sensitive derivative-based formulations that amplify sensor measurement errors, and the inability to decouple generator-internal damping from grid contributions. The proposed framework resolves these limitations through multi-scenario excitation using sensor-acquired voltage and current signals, derivative-free state consistency optimization, and physics-based regularization that enforces PH structure preservation. Complete identification of eight key parameters (*H*, *D*, $X_{d}$, $X_{q}$, $X_{d}^{\prime }$, $X_{q}^{\prime }$, $T_{do}^{\prime }$, $T_{qo}^{\prime }$) is achieved with errors ranging from 1.26% to 9.10%, and validation confirms RMS rotor angle errors below 1.2° and speed errors below 0.15%, demonstrating suitability for transient stability analysis, passivity-based control design, and oscillation damping assessment.

## 1. Introduction

The rapid transition of modern power systems toward high penetrations of renewable energy resources and inverter-based generation (IBG) has fundamentally altered grid dynamics [1]. This transformation is further accelerated by the expansion of distributed renewable generation, microgrid networks, and offshore energy systems, including emerging offshore and underwater renewables, energy harvesting infrastructures, and solar-driven energy platforms operating in marine environments [2,3]. Traditional modeling in power systems usually simplifies the power system equipment with the assumptions of balanced three-phase voltages, purely sinusoidal waveforms at a fundamental frequency, quasi-steady-state stator dynamics, and so on. These assumptions allow us to use low-order simplified models for equipment and get high-fidelity results. Unlike traditional synchronous generation, inverter-dominated resources introduce fast control loops, reduced inertia, and strong nonlinear interactions, such as Phase-Locked Loop (PLL) synchronization dynamics, fast-switching electromagnetic transients, and non-sinusoidal voltage distortions, which challenge traditional assumptions underlying power system stability analysis [4,5]. Higher-order synchronous machine models—such as the ninth-order model that includes stator flux transients and damper winding equations—can capture these effects more accurately. Even when simplified to a seventh-order model, the Port-Hamiltonian (PH) structure naturally extends to these higher-order dynamics. This allows the model to include the damper windings without losing its passivity and energy-balance properties [6]. As a result, power grids increasingly operate in regimes characterized by structural uncertainty, heterogeneous dynamics, and limited model transparency. These trends demand stability analysis frameworks that are not only robust to nonlinear behavior and large disturbances but also capable of preserving physical interpretability across interconnected subsystems [7].

Lyapunov-based stability analysis remains one of the most rigorous tools for certifying nonlinear system stability [7]. By using energy-like Lyapunov functions, such methods provide formal guarantees of transient and asymptotic stability without reliance on local linearization. In power systems, energy-based approaches have long played a central role in transient stability assessment, yet their applicability is often limited by restrictive assumptions, lossless models, or difficulty in constructing suitable Lyapunov functions for realistic systems with dissipation and control interactions [8]. The PH framework addresses these limitations by offering a physically grounded system representation in which energy storage, dissipation, and power-preserving interconnections are explicitly encoded in the model structure. Within this formalism, the Hamiltonian model naturally serves as a Lyapunov function candidate, rendering passivity and stability properties intrinsic to the system description rather than externally imposed [9].

PH modeling has been successfully applied to a broad class of electromechanical and power system components, including synchronous generators, multi-machine systems, and power-electronics-interfaced networks. By representing generators as port-controlled Hamiltonian systems, excitation, damping, and network interactions can be systematically incorporated while preserving modularity and physical consistency [10,11]. Moreover, energy-dissipating control strategies designed within the PH framework have demonstrated strong transient stability guarantees for large-scale multi-machine systems under severe disturbances [12]. These results highlight the suitability of PH representations for nonlinear stability analysis in increasingly complex grid environments.

Despite these advantages, a critical barrier limits the practical deployment of PH-based stability analysis in modern power systems: the lack of reliable, high-fidelity models. Equipment vendors increasingly withhold detailed internal models of converters, excitation systems, and control architectures due to intellectual property constraints [13]. Even for conventional synchronous generators, parameter uncertainty arising from aging equipment, operating-point variability, and undocumented control modifications undermines the accuracy of physics-based models. Consequently, systematic parameter identification methods compatible with the PH structure are urgently needed [9]. While recent work has explored learning and reconstruction of PH systems from data using machine learning, symbolic computation, and geometric methods, these approaches have not yet been fully integrated into power-system-specific stability and control analysis [14]. From the perspective of a synchronous generator, this gap is particularly consequential. Synchronous machines continue to provide the dominant source of inertia, fault current, and grid-forming capability in many transmission systems, even as inverter-based resources proliferate. Accurate generator models are therefore essential for analyzing nonlinear grid decoupling, inter-area oscillations, and transient stability margins. PH representations of synchronous generators explicitly capture the coupling between mechanical, electromagnetic, and network dynamics, enabling structure-preserving decomposition and passivity-based analysis [11]. However, without data-driven identification of Hamiltonian parameters, damping matrices, and port interconnection terms, these benefits remain largely theoretical.

Recent advances in learning Port-Hamiltonian representations from data have begun to address the challenge of recovering physically consistent models without full first-principles knowledge. In [15] the authors demonstrate that the port-based modular structure of PH systems can be exploited to learn separable subsystem representations from noisy input–output measurements, enabling learned dynamics to be transferred across different system configurations and even across physical domains. Developing on this work, ref. [16] establishes rigorous learnability conditions for linear PH systems, showing that the parameter complexity needed to replicate PH dynamics scales as O(n) rather than $O(n^{2})$, and that canonical PH systems can be uniquely identified from input–output data up to state initialization. These results demonstrate that physically meaningful PH structures are not only learnable in principle, but that the geometry of the PH parameter space can be explicitly characterized in ways that accelerate convergence and guarantee structural consistency.

Motivated by these challenges, this paper proposes a data-driven parameter identification framework for synchronous generators formulated within the PH paradigm. By combining measurement-based identification with energy-consistent model structures, the proposed approach aims to recover physically interpretable PH representations directly from operational data. This enables nonlinear grid decoupling and Lyapunov-based stability analysis without reliance on proprietary vendor models. In doing so, the work bridges the gap between data-driven modeling and energy-based stability theory, providing a pathway toward robust, transparent, and scalable stability assessment in future power systems.

## 2. Literature Review

Energy-based modeling has played a foundational role in power system stability analysis, particularly for transient and large-disturbance scenarios. Classical energy function methods aim to assess stability by evaluating system trajectories relative to an energy landscape, offering an alternative to computationally expensive time-domain simulations. However, for realistic multi-machine systems with damping, control inputs, and network losses, constructing valid global energy or Lyapunov functions remains challenging. Algorithmic approaches based on sum-of-squares programming and semidefinite optimization have been proposed to systematically construct Lyapunov functions for polynomialized power system models, yet these methods often suffer from scalability issues and conservatism when applied to large networks [8].

Since the Hamiltonian function of a PH system satisfies a dissipation inequality by construction, it serves as a natural Lyapunov candidate without requiring the energy function derivations that complicate classical transient stability methods [17]. In converter-dominated grid transient energy functions derived from virtual synchronous machine dynamics are used to certify stability margins and compute critical clearing times directly [18]. These results highlight that energy-consistent system representations are prerequisites for reliable stability certification in modern power systems.

Alternative formulations based on dissipativity and small-gain theory have been explored to analyze stability in large-scale interconnected power systems. These approaches provide sufficient stability conditions under uncertainty and delays, but typically abstract away the underlying physical energy flows, limiting their interpretability and direct applicability to controller synthesis [19]. Collectively, these works underscore the need for modeling frameworks that embed energy structure directly into system dynamics. PH theory provides a unifying framework for modeling multi-physical systems by explicitly representing energy storage, dissipation, and power-conserving interconnections. Foundational developments establish passivity and Lyapunov stability as intrinsic properties of PH systems, extending classical Hamiltonian mechanics to open and dissipative systems [9]. This framework has been further generalized to accommodate constrained dynamics and differential-algebraic equations, which naturally arise in networked systems.

In particular, linear and nonlinear PH descriptor systems have been developed to preserve energy balance and passivity in the presence of algebraic constraints. These formulations enable systematic regularization and equivalence transformations while maintaining physical interpretability, making them suitable for large-scale interconnected systems such as power networks [9,20]. Structure-preserving discretization schemes further ensure that numerical simulations retain the stability and passivity properties of the continuous-time models, which are essential for reliable analysis and control design in practical settings. Beyond theoretical developments, PH modeling has been applied to a wide range of electromechanical systems, providing insight into stability and control through energy-based reasoning [21]. Applications to electrical machines and induction motors demonstrate how PH formulations unify electromagnetic and mechanical dynamics while enabling passivity-based control design. Similar energy-based formulations have been employed in power electronics and networked systems, highlighting the modularity and scalability of the PH paradigm.

In the context of power networks, PH representations facilitate structure-preserving decomposition of complex systems into interconnected subsystems. This modular viewpoint supports decentralized analysis and control, as well as robustness assessment under interconnection uncertainties. However, most existing PH-based power system studies rely on detailed first-principles models and assume precise knowledge of system parameters, limiting their applicability in data-scarce or vendor-restricted environments [21,22].

A substantial body of work has investigated transient stability enhancement through energy-dissipating and passivity-based control strategies. Hybrid control schemes and excitation control laws designed within energy-based frameworks have demonstrated improved damping and stability margins under large disturbances [12]. Power-electronics-enabled stabilization approaches further exploit fast control action to shape system energy and suppress unstable modes, particularly in stressed operating conditions [23]. While these approaches illustrate the effectiveness of energy-based control, they remain fundamentally model-driven: accurate knowledge of local system parameters and interconnection structure is typically assumed, and the impact of parameter uncertainty or model mismatch on stability guarantees is often not explicitly addressed.

For example, passivity-based virtual oscillator control strategies derived within a PH framework have demonstrated global asymptotic synchronization properties without requiring explicit knowledge of the full network dynamics [22]. These results underscore the effectiveness of PH-based control for converter-dominated systems. However, they still rely on accurate local models and parameters, and the focus remains on control synthesis and stability certification rather than on systematic identification of energy-consistent models from data. Parallel to developments in energy-based control, data-driven modeling has gained significant traction in power systems due to the increasing availability of high-resolution measurements and the growing opacity of vendor-supplied models. Techniques such as dynamic mode decomposition (DMD), sparse identification of nonlinear dynamical systems (SINDy), and Koopman-based methods have been applied to model converter-dominated systems and virtual synchronous machines directly from data [24].

Recent work demonstrates that optimized DMD and SINDy can accurately capture the transient and steady-state dynamics of grid-connected virtual synchronous machines, yielding reduced-order models suitable for stability assessment and control design [24]. While these approaches offer flexibility and scalability, they are inherently black-box or weakly structured and do not enforce physical energy consistency or passivity. As a result, their direct use in Lyapunov-based stability analysis remains limited.

Recent advances have begun to address the challenge of identifying PH systems from data. A notable contribution proposes a two-stage reconstruction framework that combines machine learning for graph identification with symbolic computation and geometric methods to recover Hamiltonian structure and port interconnections from observed dynamics [14]. This work demonstrates that PH structure can be inferred rather than prescribed, preserving passivity and interpretability. A notable contribution proposes a structure-preserving identification framework for nonlinear PH systems using input–state–output data and neural network parametrizations of the Hamiltonian, interconnection, and dissipation matrices [25], as well as Bayesian learning approaches incorporating physics priors [26]. While these neural network-based methods successfully yield mathematically stable PH models, they attempt to learn the entire abstract energy function and interconnection matrices from scratch across thousands of training trajectories. The resulting model acts as a black-box mapping that does not yield explicit engineering parameters (such as $H,X_{d},X_{q}^{\prime }$), drastically limiting its analytical utility for standard power system tasks like fault current calculations. Moreover, these approaches demand high computational costs for offline processing and are primarily developed for mechanical or abstract dynamical systems.

Recent advancements in high-resolution sensor data, particularly Phasor Measurement Units (PMUs), have driven modern data-driven and machine learning-assisted approaches for synchronous generator modeling. PMU-based dynamic parameter identification algorithms increasingly rely on sudden grid disturbances or ambient data to extract physical system properties. Concurrently, there are new developments in physics-informed identification strategies that embed physical constraints directly into the optimization loop to prevent non-physical parameter estimates, similar to the Port-Hamiltonian consistency approach presented in this work.

IEEE Standard 115-2019 provides comprehensive guidelines for determining synchronous machine parameters through standardized testing procedures. Clause 11 of this standard specifically addresses transient parameter identification for dynamic analysis through sudden short-circuit tests, load rejection tests, voltage recovery tests, and stationary tests for extracting direct-axis and quadrature-axis reactances and time constants [27]. While the standard’s methodology has formed the foundation for generator modeling in power system stability studies, it exhibits fundamental limitations when applied to PH modeling: (1) single-scenario testing provides limited parameter identifiability, (2) derivative-based curve fitting is sensitive to measurement noise, (3) terminal-only test conditions neglect network impedance effects and conflate generator-internal dynamics with external grid interactions, (4) lack of energy consistency enforcement may violate passivity requirements, and (5) q-axis parameter observability remains poor under balanced faults.

To address these limitations, this work proposes a three-stage data-driven identification framework for PH synchronous generator models with the following key contributions:

1. Multi-Scenario Identification: The framework introducing diverse excitation scenarios—multiple fault durations (50–200 ms), voltage steps (±10–20%), and mechanical power perturbations (±5–10%)—to ensure parameter identifiability across operational conditions.
2. Derivative-Free State Consistency: A novel optimization formulation enforces consistency between ODE-simulated internal states and algebraically reconstructed states from terminal measurements, eliminating the noise sensitivity of curve fitting methods.
3. Grid Connected Model Identification: Systematic separation of generator-internal damping from network-induced effects by identifying mechanical parameters from isolated perturbations.
4. PH Structure Preservation: Explicit enforcement of energy balance, passivity conditions, and physical realizability constraints ensures compatibility with Lyapunov-based stability analysis.
5. Physics-Constrained Global Optimization: Integration of differential evolution with physics-informed bounds and multi-start refinement enables robust parameter space exploration.

This framework enables reliable PH parameter identification from operational data. Subsequent sections detail the three-stage methodology and validate its effectiveness against actual generator parameters.

## 3. Methodology

### 3.1. Problem Formulation

The identification of synchronous generator parameters is essential for accurate power system dynamic analysis and control design. This work focuses on identifying the complete parameter set for a fourth-order PH generator model, comprising eight parameters: the mechanical inertia constant *H* (s), the damping coefficient *D* (per unit, pu), the d-axis synchronous reactance $X_{d}$ (pu), the d-axis transient reactance $X_{d}^{\prime }$ (pu), the d-axis open-circuit time constant $T_{do}^{\prime }$ (s), the q-axis synchronous reactance $X_{q}$ (pu), the q-axis transient reactance $X_{q}^{\prime }$ (pu), and the q-axis open-circuit time constant $T_{qo}^{\prime }$ (s).

The PH representation of the synchronous generator is formulated using the state vector $x={\left[\delta ,\omega ,E_{q}^{\prime },E_{d}^{\prime }\right]}^{T}$ and the Hamiltonian (total energy) function H(x) in (1):

(1) $$H\left(x\right)=H{\left(\omega -1\right)}^{2}+\frac{T_{do}^{\prime }}{2}{\left(E_{q}^{\prime }\right)}^{2}+\frac{T_{qo}^{\prime }}{2}{\left(E_{d}^{\prime }\right)}^{2},$$

where the first term represents kinetic energy, and the remaining terms represent magnetic field energy storage. The system dynamics follow the canonical PH form (2):

(2) $$\dot{x}=\left(J-R\right)\frac{\partial H}{\partial x}+gu,$$

where J is the skew-symmetric interconnection matrix, R is the positive semidefinite dissipation matrix, g is the input matrix, and u represents external inputs. For the synchronous generator (3) and (4):

(3) $$J=\left[\begin{array}{cccc} 0 & \omega_{s} & 0 & 0\\ -\omega_{s} & 0 & 0 & 0\\ 0 & 0 & 0 & 0\\ 0 & 0 & 0 & 0 \end{array}\right],\;R=\left[\begin{array}{cccc} 0 & 0 & 0 & 0\\ 0 & D/(2H) & 0 & 0\\ 0 & 0 & 1/T_{do}^{\prime } & 0\\ 0 & 0 & 0 & 1/T_{qo}^{\prime } \end{array}\right],$$

(4) $$g=\left[\begin{array}{ccc} 0 & 0 & 0\\ 1/(2H) & -1/(2H) & 0\\ -\left(X_{d}-X_{d}^{\prime }\right)/T_{do}^{\prime } & 0 & 1/T_{do}^{\prime }\\ \left(X_{q}-X_{q}^{\prime }\right)/T_{qo}^{\prime } & 0 & 0 \end{array}\right],\;u=\left[\begin{array}{c} P_{m}\\ P_{e}\\ V_{f} \end{array}\right].$$

Expanding Equation (2) yields the explicit differential equations:

(5) $$\begin{array}{cc} \frac{d\delta }{dt} & =\left(\omega -1\right)\omega_{s}, \end{array}$$

(6) $$\begin{array}{cc} \frac{d\omega }{dt} & =\frac{1}{2H}\left(P_{m}-P_{e}-D\left(\omega -1\right)\right), \end{array}$$

(7) $$\begin{array}{cc} \frac{dE_{q}^{\prime }}{dt} & =\frac{1}{T_{do}^{\prime }}\left(V_{f}-E_{q}^{\prime }-\left(X_{d}-X_{d}^{\prime }\right)I_{d}\right), \end{array}$$

(8) $$\begin{array}{cc} \frac{dE_{d}^{\prime }}{dt} & =\frac{1}{T_{qo}^{\prime }}\left(-E_{d}^{\prime }+\left(X_{q}-X_{q}^{\prime }\right)I_{q}\right), \end{array}$$

where δ is the rotor angle (rad), ω is the rotor speed (pu), $\omega_{s}=2\pi f_{0}$ is the synchronous frequency (rad/s) with $f_{0}=50\mathrm{Hz}$, $P_{m}$ is the mechanical power (pu), $P_{e}$ is the electrical power (pu), $V_{f}$ is the field voltage (pu), and $I_{d}$, $I_{q}$ are the d-axis and q-axis stator currents (pu), respectively.

The interconnection matrix J preserves energy exchange between mechanical and electrical subsystems, while the dissipation matrix R captures irreversible energy losses through mechanical damping and field winding resistance. The algebraic equations relating terminal voltage and internal fluxes are (9)–(13):

(9) $$\begin{array}{cc} V_{d} & =V_{t}\sin\delta , \end{array}$$

(10) $$\begin{array}{cc} V_{q} & =V_{t}\cos\delta , \end{array}$$

(11) $$\begin{array}{cc} I_{d} & =\frac{E_{q}^{\prime }-V_{q}}{X_{d}^{\prime }}, \end{array}$$

(12) $$\begin{array}{cc} I_{q} & =\frac{V_{d}-E_{d}^{\prime }}{X_{q}^{\prime }}, \end{array}$$

(13) $$\begin{array}{cc} P_{e} & =V_{d}I_{d}+V_{q}I_{q}, \end{array}$$

where $V_{t}$ is the terminal voltage magnitude (pu).

### 3.2. IEEE 115 Standard Method for Transient Parameter Identification

The IEEE Standard 115-2019 provides the established methodology for determining synchronous generator parameters through sudden short-circuit tests. The theoretical foundation of this approach is based on the response of the generator to a three-phase terminal short circuit applied from an initially unloaded or lightly loaded condition.

#### 3.2.1. Theoretical Basis of IEEE 115 Method

When a synchronous generator experiences a sudden three-phase short circuit at its terminals, the short-circuit current exhibits a characteristic decay pattern that can be decomposed into multiple exponential components [27]. According to IEEE 115-2019, the symmetrical component of the short-circuit current can be expressed as (14):

(14) $$i_{sc}\left(t\right)=E\left[\frac{1}{X_{d}^{\prime \prime }}\exp\left(-\frac{t}{T_{d}^{\prime \prime }}\right)+\frac{1}{X_{d}^{\prime }}\exp\left(-\frac{t}{T_{d}^{\prime }}\right)+\frac{1}{X_{d}}\right],$$

where *E* is the internal voltage prior to the fault (pu), $X_{d}^{\prime \prime }$ is the d-axis subtransient reactance (pu), $X_{d}^{\prime }$ is the d-axis transient reactance (pu), $X_{d}$ is the d-axis synchronous reactance (pu), $T_{d}^{\prime \prime }$ is the d-axis subtransient short-circuit time constant (s), and $T_{d}^{\prime }$ is the d-axis transient short-circuit time constant (s).

For the time scales relevant to transient stability analysis (typically 1 to 10 s), the subtransient component decays rapidly (within the first few cycles) and can be neglected. The simplified two-component model becomes (15):

(15) $$i_{sc}\left(t\right)=E\left[\frac{1}{X_{d}^{\prime }}\exp\left(-\frac{t}{T_{d}^{\prime }}\right)+\frac{1}{X_{d}}\right].$$

The parameter extraction procedure involves fitting the measured short-circuit current envelope to this exponential model. The transient reactance $X_{d}^{\prime }$ and transient short-circuit time constant $T_{d}^{\prime }$ are directly obtained from this fit. The open-circuit time constant $T_{do}^{\prime }$, which appears in the flux dynamics Equation (7), is then calculated using the IEEE 115 conversion Formula (16):

(16) $$T_{do}^{\prime }=T_{d}^{\prime }\;\cdot \;\frac{X_{d}}{X_{d}^{\prime }}.$$

This relationship arises from the fact that the short-circuit time constant represents the decay with a short-circuited armature, while the open-circuit time constant represents the decay with an open-circuited armature.

Similarly, for the q-axis, the current response during and after the fault can be analyzed to extract $X_{q}^{\prime }$ and $T_{q}^{\prime }$, with the open-circuit time constant given by (17):

(17) $$T_{qo}^{\prime }=T_{q}^{\prime }\;\cdot \;\frac{X_{q}}{X_{q}^{\prime }}.$$

#### 3.2.2. Implementation of IEEE 115 Method

The practical implementation of the IEEE 115 method in this work utilizes multiple fault scenarios with varying durations (50, 100, 150, and 200 milliseconds) to enhance robustness. For each fault scenario, the following procedure is applied:

1. Detection of fault initiation and clearing times based on terminal voltage magnitude $V_{t}<0.3V_{pf}$ (pf: prefault);
2. Extraction of the current magnitude time series I(t) from fault initiation to several seconds post-clearing;
3. Formulation of the optimization problem to fit Equation (15) to the measured data;
4. Application of global optimization using the differential evolution algorithm with bounds:
  (18) $$\begin{array}{cc} 0.7E_{pf} & \leq E\leq 1.3E_{pf}\\ 0.8X_{d,ref} & \leq X_{d}\leq 1.2X_{d,ref}\\ 0.1 & \leq X_{d}^{\prime }\leq 0.6\\ 0.2 & \leq T_{d}^{\prime }\leq 2.5; \end{array}$$
5. Calculation of $T_{do}^{\prime }$ using Equation (16).

The cost function minimized in the optimization is the weighted sum of squared errors (19):

(19) $$J=\sum_{i=1}^{N}w_{i}{\left[I_{meas}\left(t_{i}\right)-I_{model}\left(t_{i}\right)\right]}^{2},$$

where $w_{i}$ represents weights proportional to the fault duration (longer faults receive higher weights due to a better signal-to-noise ratio), and *N* is the total number of data points across all fault scenarios.

For q-axis identification, the q-axis current component $I_{q}\left(t\right)$ is extracted and fitted to (20):

(20) $$I_{q}\left(t\right)=E\left[\frac{1}{X_{q}^{\prime }}\exp\left(-\frac{t}{T_{q}^{\prime }}\right)+\frac{1}{X_{q}}\right],$$

with optimization bounds:

(21) $$\begin{array}{cc} 0.7E_{pf} & \leq E\leq 1.3E_{pf}\\ 0.8X_{q,ref} & \leq X_{q}\leq 1.2X_{q,ref}\\ 0.7 & \leq X_{q}^{\prime }\leq 1.1\\ 0.045 & \leq T_{q}^{\prime }\leq 0.15. \end{array}$$

### 3.3. Proposed Identification Framework

To address the limitations of the IEEE 115 method and obtain the complete eight-parameter set while accounting for grid coupling, this work develops a novel three-stage identification framework that synergistically combines complementary identification techniques.

The proposed framework recognizes that different parameter groups exhibit distinct observability characteristics under different operating conditions and test scenarios. Rather than attempting to identify all eight parameters simultaneously from a single scenario type (as in conventional approaches), the framework strategically partitions the identification problem into three stages:

1. Stage 1 (Mechanical Parameters): Exploits electromechanical oscillations in torque response scenarios to identify *H* and *D*, with a novel differential approach to decouple grid damping effects.
2. Stage 2 (Steady-State Reactances):Utilizes steady-state voltage–current relationships from multiple operating points to identify $X_{d}$ and $X_{q}$ with high accuracy.
3. Stage 3 (Transient Parameters): Employs a derivative-free state consistency method across all available scenarios to identify $X_{d}^{\prime }$, $X_{q}^{\prime }$, $T_{do}^{\prime }$, and $T_{qo}^{\prime }$.

To ensure complete identifiability, driving scenarios were explicitly designed to target complementary parameter groups based on their physical orientation. Standard short-circuit faults and voltage perturbations primarily demand massive reactive power support, predominantly excite the *d*-axis currents ($I_{d}$). This effectively reveals the *d*-axis parameters ($X_{d}^{\prime },T_{do}^{\prime }$). On the other hand, active power dynamics ($P_{e}=V_{d}I_{d}+V_{q}I_{q}$) are heavily dominated by the *q*-axis current $I_{q}$ under typical load angles. By introducing targeted mechanical torque steps, the framework deliberately forces the rotor to accelerate and swing its load angle. This rapid deviation violently shifts the active power balance, generating massive transient perturbations in $I_{q}$ that explicitly excite the interconnected *q*-axis internal voltage state $E_{d}^{\prime }$. Thus, the torque scenarios push the $X_{q}^{\prime }$ and $T_{qo}^{\prime }$ parameters out of their steady-state zone, and make them more observable.

The proposed optimization framework has three stages. Stage 1 utilizes exponential curve fitting (Levenberg–Marquardt least squares) on the detected oscillation peaks of the speed signal to analytically extract the mechanical bounds. Stage 2 employs bounded linear regression via the L-BFGS-B algorithm on steady-state measurements to calculate direct closed-form slopes for the reactances. Stage 3 performs a derivative-free state consistency global search using differential evolution (population size 12, max 300 generations, Latin-hypercube initialization) followed by a multi-start L-BFGS-B local refinement to guarantee a robust minimum while applying physics-informed penalties. The detailed pseudo-code of the three-stage process is outlined in Algorithm 1.
**Algorithm 1** Three-stage data-driven PH parameter identification  1:**procedure** Stage1($S_{\mathrm{torque}}$)                             ▹ Mechanical parameters: *H*, *D*  2:      **for** each dampingless/normal pair $\left(S_{\mathrm{dl},i},S_{\mathrm{norm},i}\right)$ **do**  3:             Fit exponential envelope to ω(t) peaks →$\left(\zeta ,\omega_{n}\right)$ per scenario  4:             $D_{\mathrm{gen},i}\leftarrow 4H_{\mathrm{init}}\;\left(\zeta_{\mathrm{norm}}\;\omega_{n,\mathrm{norm}}-\zeta_{\mathrm{dl}}\;\omega_{n,\mathrm{dl}}\right)$  5:      **end for**  6:      $D_{\mathrm{id}}\leftarrow \mathrm{mean}\left\{D_{\mathrm{gen},i}\right\}$  7:      $H\left(t_{k}\right)\leftarrow \frac{P_{m}-P_{e}-D_{\mathrm{id}}\left(\omega -1\right)}{2\dot{\omega }}$,   $\forall \;|\dot{\omega }|>0.001$  8:      **return** $H_{\mathrm{id}}\leftarrow \mathrm{median}\left\{H\left(t_{k}\right)\right\}$,   $D_{\mathrm{id}}$  9:**end procedure**10:**procedure** Stage2($S_{V}$,$S_{\mathrm{torque}}$)                       ▹ Steady-state reactances: $X_{d}$, $X_{q}$11:      Fit $V_{q}=-X_{d}I_{d}+E_{q0}^{\prime }$ to steady-state PT/CT sensors data via bounded L-BFGS-B12:     Fit Vd=−XqIq+Ed0′ to steady-state CT sensor data via polynomial regression13:      **return** $X_{d}$,   $X_{q}$14:**end procedure**15:**procedure** Stage3($S_{\mathrm{all}}$,$X_{d}$,$X_{q}$)          ▹ Transient parameters: $X_{d}^{\prime }$, $X_{q}^{\prime }$, $T_{do}^{\prime }$, $T_{qo}^{\prime }$16:      Minimize state consistency objective J(θ) over all scenarios:$$J\left(\theta \right)=\frac{1}{N}\sum_{s}w_{s}\sum_{k}\;\left[{\left(E_{q,k}^{\prime \;\mathrm{sim}}-E_{q,k}^{\prime \;\mathrm{recon}}\right)}^{2}+{\left(E_{d,k}^{\prime \;\mathrm{sim}}-E_{d,k}^{\prime \;\mathrm{recon}}\right)}^{2}\right]+\lambda_{\mathrm{pen}}\;P\left(\theta \right)$$17:      Global: differential evolution (pop = 12, 300 gen., Latin-hypercube init.)18:      Local: multi-start L-BFGS-B with physics constraints ($X_{d}^{\prime }<X_{d}$, $X_{q}^{\prime }<X_{q}$, $T_{do}^{\prime }>T_{qo}^{\prime }$)19:      **return** $\theta^{*}=\arg\min J$:   $X_{d}^{\prime }$,$X_{q}^{\prime }$,$T_{do}^{\prime }$,$T_{qo}^{\prime }$20:**end procedure**21:**Output:** $\left\{H,\;D,\;X_{d},\;X_{q},\;X_{d}^{\prime },\;X_{q}^{\prime },\;T_{do}^{\prime },\;T_{qo}^{\prime }\right\}$

This staged approach offers several advantages: (1) parameter decoupling reduces correlation and improves numerical conditioning, (2) each stage uses scenarios optimally suited for the target parameters, (3) inter-stage constraints ensure global consistency, and (4) the framework systematically addresses grid coupling effects that confound conventional methods.

#### 3.3.1. Stage 1: Mechanical Parameter Identification with Grid Decoupling

The mechanical parameters *H* and *D* are identified from torque step response data. The swing Equation (6) can be rearranged to (22):

(22) $$2H\frac{d\omega }{dt}=P_{m}-P_{e}-D\left(\omega -1\right).$$

The challenge lies in separating the generator damping $D_{gen}$ from the grid-contributed damping $D_{grid}$. This work introduces a differential damping identification method that exploits paired simulation scenarios.

The method utilizes two sets of torque step scenarios:

1. Dampingless scenarios: Generator damping disabled in simulation ($D_{gen}=0$).
2. Normal scenarios: Generator damping enabled with identical torque disturbances.

For each pair of scenarios with matching torque levels $\Delta P_{m}$, the damping coefficient is extracted by analyzing the oscillation decay characteristics. The speed response following a torque disturbance exhibits damped oscillations that can be approximated as (23):

(23) $$\omega \left(t\right)-\omega_{ss}=A_{0}e^{-\alpha t}\cos\left(\omega_{d}t+\phi \right),$$

where $\omega_{ss}$ is the steady-state speed, $A_{0}$ is the initial amplitude, α is the decay rate, $\omega_{d}$ is the damped oscillation frequency, and ϕ is the phase angle.

By identifying the peaks of the oscillation and fitting an exponential envelope $A_{0}e^{-\alpha t}$ to these peaks, the damping ratio is calculated as (24):

(24) $$\zeta =\frac{\alpha }{\sqrt{\alpha^{2}+\omega_{d}^{2}}}.$$

The undamped natural frequency is (25):

(25) $$\omega_{n}=\frac{\omega_{d}}{\sqrt{1-\zeta^{2}}}.$$

The total damping coefficient is then (26):

(26) $$D_{total}=4\zeta H\omega_{n},$$

where the factor of 4 accounts for the per unit system and the definition of damping in the swing equation. The factor can be derived by considering the standard second-order system equation $2H\ddot{\omega }+D\dot{\omega }=0$, which in normalized form gives $\ddot{\omega }+2\zeta \omega_{n}\dot{\omega }+\omega_{n}^{2}\omega =0$, leading to $D=4\zeta H\omega_{n}$.

For the dampingless (dl) scenario (27):

(27) $$D_{grid}=4\zeta_{dl}H\omega_{n,dl}.$$

For the normal scenario (28):

(28) $$D_{total}=4\zeta_{normal}H\omega_{n,normal}.$$

The generator damping is isolated through subtraction (29):

(29) $$D_{gen}=D_{total}-D_{grid}.$$

This differential approach effectively decouples the grid influence from the generator characteristic. Multiple scenario pairs are analyzed, and the results are averaged to improve robustness (30):

(30) $$D_{id}=\frac{1}{N_{pairs}}\sum_{i=1}^{N_{pairs}}D_{gen,i}.$$

Once *D* is determined, the inertia constant *H* is identified by solving Equation (22). Combining data from all torque scenarios (both dampingless and normal), the equation is discretized as (31):

(31) $$H=\frac{P_{m}\left(t_{k}\right)-P_{e}\left(t_{k}\right)-D\left(\omega \left(t_{k}\right)-1\right)}{2{\left(\frac{d\omega }{dt}\right)}_{t_{k}}}.$$

The time derivative $\frac{d\omega }{dt}$ is computed using numerical differentiation with appropriate filtering. Points where $|\frac{d\omega }{dt}|<0.001$ are excluded to avoid numerical instability. The identified values $H(t_{k})$ are computed at all valid time points, and the median is taken to obtain a robust estimate (32):

(32) $$H_{id}=\mathrm{median}\{H\left(t_{k}\right):|\frac{d\omega }{dt}{|}_{t_{k}}>0.001,\;1.0<H\left(t_{k}\right)<10.0\}.$$

The bounds in Equation (32) are based on physical feasibility for typical synchronous generators.

#### 3.3.2. Stage 2: Steady-State Reactance Identification

The steady-state synchronous reactances $X_{d}$ and $X_{q}$ are identified from voltage change scenarios. These scenarios involve step changes in the external grid voltage, which alter the terminal voltage and consequently the steady-state operating point while maintaining constant mechanical input and field excitation.

For d-axis identification, the steady-state relationship from Equations (7) and (11) gives (33):

(33) $$\frac{dE_{q}^{\prime }}{dt}=0\Rightarrow V_{f}=E_{q}^{\prime }+\left(X_{d}-X_{d}^{\prime }\right)I_{d}.$$

At a steady state, $E_{q}^{\prime }$ can be expressed in terms of terminal voltage and angle. For the identification, the q-axis voltage–current relationship is utilized (34):

(34) $$V_{q}=E_{q}^{\prime }-X_{d}^{\prime }I_{d}.$$

Combining steady-state conditions and Park transformation, the d-axis voltage is related to $I_{d}$ through (35):

(35) $$V_{q}=-X_{d}I_{d}+E_{q0}^{\prime },$$

where $E_{q0}^{\prime }$ is a constant related to the field excitation. Multiple voltage scenarios with different terminal voltages $V_{t}$ are simulated, and the steady-state values (taken from the final 20% of the simulation) of $I_{d}$ and $V_{q}=V_{t}\cos\delta$ are extracted. A linear regression is performed to fit Equation (35), with the slope providing $X_{d}$ (36):

(36) $$X_{d}=-\frac{\partial V_{q}}{\partial I_{d}}.$$

The optimization problem is formulated as (37):

(37) $$\min_{X_{d},E_{q0}^{\prime }}\sum_{j=1}^{N_{scenarios}}{\left[V_{q,j}-\left(-X_{d}I_{d,j}+E_{q0}^{\prime }\right)\right]}^{2},$$

subject to physical bounds $1.0\leq X_{d}\leq 5.0$ and $0.5\leq E_{q0}^{\prime }\leq 2.0$. This bounded regression problem is solved using the Limited-memory Broyden–Fletcher–Goldfarb–Shanno with Bounds (L-BFGS-B) algorithm.

Similarly, for q-axis identification, the relationship (38):

(38) $$V_{d}=X_{q}I_{q}+E_{d0}^{\prime }$$

is used, where $V_{d}=V_{t}\sin\delta$. However, voltage changes primarily affect the d-axis dynamics. Therefore, torque step scenarios are additionally utilized for q-axis identification, as changes in mechanical power alter the power angle δ and consequently $I_{q}$. The steady-state values of $I_{q}$ and $V_{d}$ from multiple scenarios are fitted to Equation (38), yielding (39):

(39) $$X_{q}=\frac{\partial V_{d}}{\partial I_{q}}.$$

#### 3.3.3. Stage 3: State Consistency-Based Transient Parameter Identification

The transient parameters $X_{d}^{\prime }$, $X_{q}^{\prime }$, $T_{do}^{\prime }$, and $T_{qo}^{\prime }$ are identified using a novel derivative-free state consistency method that avoids the numerical differentiation challenges inherent in conventional approaches.

The PH generator model provides two independent ways to determine the internal flux states $E_{q}^{\prime }$ and $E_{d}^{\prime }$:

1. Dynamic evolution via ODEs: The differential Equations (7) and (8) describe how the internal states evolve over time given the inputs (field voltage $V_{f}$, currents $I_{d}$, $I_{q}$) and parameters.
2. Algebraic reconstruction: From the algebraic Equations (11) and (12), the internal states can be reconstructed directly from measured terminal quantities as (40) and (41):
  (40) $$\begin{array}{cc} E_{q}^{\prime recon}\left(t\right) & =V_{q}\left(t\right)+X_{d}^{\prime }I_{d}\left(t\right), \end{array}$$
  (41) $$\begin{array}{cc} E_{d}^{\prime recon}\left(t\right) & =V_{d}\left(t\right)-X_{q}^{\prime }I_{q}\left(t\right). \end{array}$$

For the correct parameter values, these two representations must be consistent: the states evolved through the ODEs should match the algebraically reconstructed states at all times.

## 4. Simulation Framework and Results

All identification procedures are implemented using data generated from DIgSILENT PowerFactory. The test system consists of a 1000 MVA, 20 kV synchronous generator connected to an IEEE 14-bus [28] test network through a step-up transformer. The generator model in PowerFactory is configured as a detailed fourth-order two-axis model.

Each measured quantity corresponds to a specific sensor type that would be deployed at the generator terminals or on the rotor shaft in practice. The terminal voltage magnitude $V_{t}$ is acquired via a voltage transducer (equivalent to a potential transformer, PT), modeled as the bus-voltage output variable m:u1:bus1. The short-circuit current magnitude $I_{sc}$ and its d/q-axis decomposition ($I_{d}$, $I_{q}$) are obtained through current transducers (equivalent to current transformers, CTs) combined with a Park-transformation signal-processing block, corresponding to the PowerFactory variables m:i1:bus1, c:id, and c:iq. The rotor speed ω is measured by a shaft-speed sensor (equivalent to a digital encoder or tachometer), mapped to s:speed. The power angle δ is derived from the voltage phasor angle, as would be supplied by a Phasor Measurement Unit (PMU) in a real installation, and is available as c:fi. The field voltage $V_{f}$ is read directly from the excitation-system model via s:ve. All variables are sampled at a uniform time step of Δt=1 ms, consistent with high-bandwidth digital fault recorders and PMU-grade measurement systems.

The scenario generation is automated through a Python interface to PowerFactory. For each scenario type, the following protocol is implemented:

1. Initialize the system to the steady-state operating point (0.8 per unit active power output, 1.0 per unit terminal voltage);
2. Apply the specified disturbance (torque step, voltage change, or fault) at t=1.0 s;
3. Simulate for 30 s with time step Δt=0.001 s;
4. Record time-series data including: rotor angle δ(t), rotor speed ω(t), d-axis current $I_{d}\left(t\right)$, q-axis current $I_{q}\left(t\right)$, terminal voltage magnitude $V_{t}\left(t\right)$, terminal voltage angle $\theta_{t}\left(t\right)$, mechanical torque $T_{m}\left(t\right)$, electrical power $P_{e}\left(t\right)$, and reactive power $Q_{e}\left(t\right)$.

The generated data is exported to CSV format and processed by the identification algorithms implemented in Python 3.12.3 using NumPy 1.26.4, SciPy 1.11.4 and Pandas 2.1.4 libraries.

The complete scenario set comprises:

- Dampingless torque scenarios: Two scenarios with $\Delta P_{m}=+0.10$ and −0.10 per unit, with generator damping disabled;
- Normal torque scenarios: Five scenarios with $\Delta P_{m}=+0.05,+0.10,+0.15,-0.05,-0.10$ per unit;
- Voltage source change scenarios: Four scenarios with terminal voltage setpoints of 1.40,1.20,0.80,0.60 per unit;
- Three-phase fault scenarios: Four scenarios with fault durations of 50,100,150 and 200 ms.

This comprehensive dataset enables robust parameter identification across multiple operating conditions and disturbance types.

**Figure 1:**
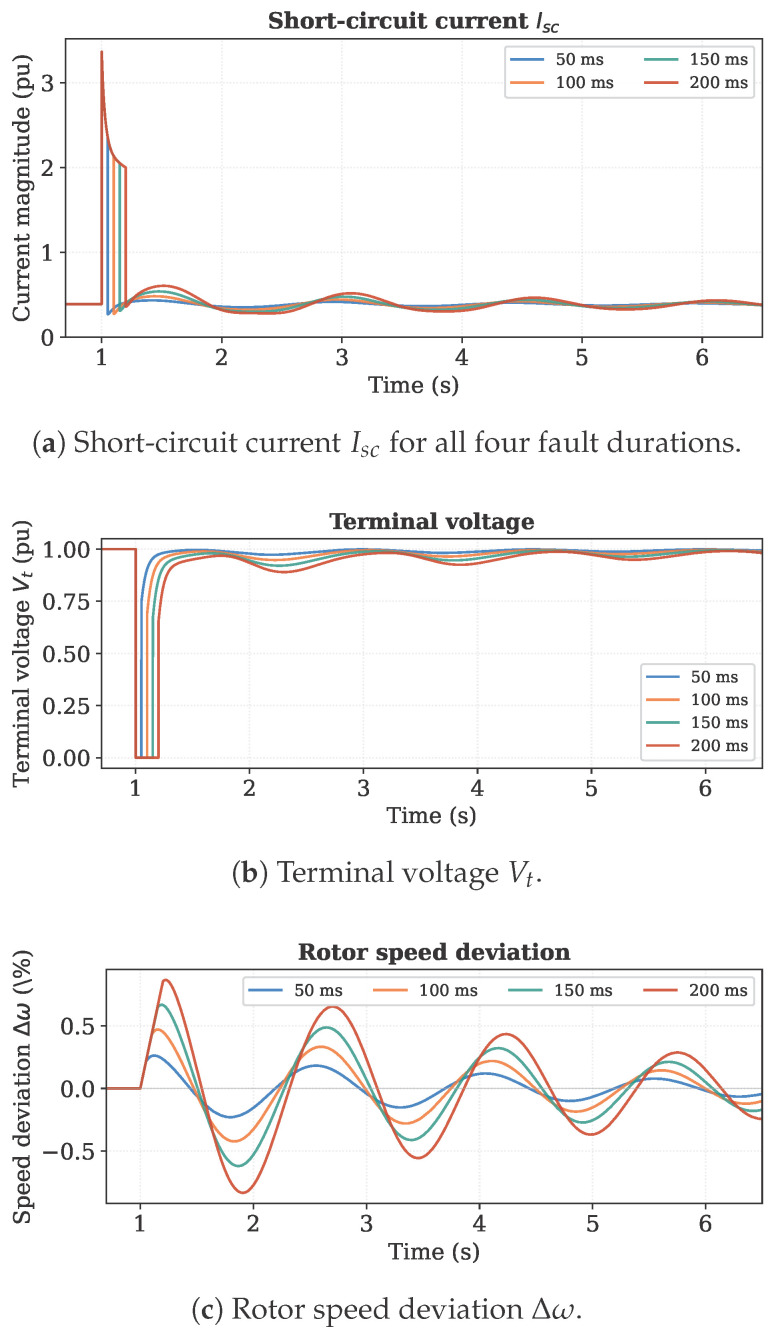
Simulated short-circuit data for four fault durations (50, 100, 150, and 200 ms). Visible post-fault electromechanical oscillations confirm the need for a multi-scenario, grid-aware identification framework.

Figure 1 presents the complete short-circuit current $I_{sc}$ time series for all four fault durations (50, 100, 150, and 200 ms), each shown in a distinct color. The figure also includes the supporting sensor signals directly exploited by the proposed identification framework: terminal voltage $V_{t}$, rotor speed deviation Δω, and the d- and q-axis stator currents $I_{d}$ and $I_{q}$. First, the post-fault current exhibits damped electromechanical oscillations that a simple exponential model cannot reproduce; this behavior arises directly from the generator’s grid coupling and is a key motivation for the derivative-free state consistency method proposed in Stage 3. Second, the severity of the transient response increases with fault duration, providing richer signal content for the optimization and confirming the benefit of using multiple scenarios simultaneously.

### 4.1. Actual Generator Parameters

The synchronous generator model in PowerFactory is configured with the parameters shown in Table 1, which serve as the reference values for assessing identification accuracy.

#### 4.1.1. Stage 1: Mechanical Parameters

The differential damping method was applied to the paired torque scenarios. For the scenario pair with $\Delta P_{m}=+0.10$ pu:

- Dampingless scenario (S11 dampingless torque +0.10pu): $D_{grid}=0.150$ pu;
- Normal scenario (S12 torque + 0.10 pu): $D_{total}=0.570$ pu;
- Identified generator damping: $D_{gen}=0.570-0.150=0.420$ pu.

The oscillation decay analysis for the dampingless scenario yielded a damping ratio $\zeta_{dl}=0.0123$ and natural frequency $\omega_{n,dl}=3.48$ rad/s, leading to $D_{grid}=4\times 0.0123\times 3.48\times 3.48=0.150$ pu using Equation (26).

For the normal scenario, $\zeta_{normal}=0.0416$ and $\omega_{n,normal}=3.45$ rad/s, giving $D_{total}=4\times 0.0416\times 3.48\times 3.45=0.570$ pu.

Repeating this analysis for the negative torque step pair ($\Delta P_{m}=-0.10$ pu) yielded $D_{gen}=0.419$ pu. The average over the two pairs gives (42):

(42) $$D_{id}=\frac{0.420+0.419}{2}=0.4198\;\mathrm{pu}.$$

This represents an error of (0.4198−0.400)/0.400=+4.95% relative to the actual value of 0.400 pu.

Using the identified D=0.4198 pu in the swing equation analysis across all seven torque scenarios (both dampingless and normal), the inertia constant was determined by applying Equation (31) to 14,237 data points (after filtering for $|\frac{d\omega }{dt}|>0.001$). The median of the computed *H* values is 3.436s, corresponding to an error of (3.4361−3.480)/3.480=−1.26%. This demonstrates the effectiveness of the differential damping approach in decoupling the generator mechanical characteristics from grid influences (Table 2).

#### 4.1.2. Stage 2: Steady-State Reactances

The four voltage source change scenarios (1.40, 1.20, 0.80, 0.60 pu) provided steady-state operating points spanning a wide range of terminal voltages. The steady-state values extracted from the final 20% of each simulation are in Table 3.

Linear regression according to Equation (37) yields (43):

(43) $$X_{d}=2.457\;\mathrm{pu},\;E_{q0}^{\prime }=0.938\;\mathrm{pu},$$

with coefficient of determination $R^{2}=0.9987$, indicating excellent linear fit quality. The identified $X_{d}$ exhibits an error of (2.4574−2.540)/2.540=−3.25%.

For q-axis identification, the seven torque step scenarios provide variations in the power angle and consequently in $I_{q}$ and $V_{d}$, as shown in Table 4.

The linear regression, with $R^{2}=0.9923$, gives (44):

(44) $$X_{q}=2.191\;\mathrm{pu},\;E_{d0}^{\prime }=-0.401\;\mathrm{pu}.$$

As shown in Table 5, the identified $X_{q}$ has an error of (2.1907−2.410)/2.410=−9.10%, which, while larger than the d-axis error, provides physical consistency with the operating characteristics of the generator.

#### 4.1.3. Stage 3: Transient Parameters Using State Consistency Method

The optimization employed multi-start differential evolution with L-BFGS-B local refinement, using the IEEE 115-based initial estimates as prior guidance with weight $\lambda_{prior}=0.2$. The steady-state reactances were fixed to the Stage 2 values: $X_{d}^{fixed}=2.4574$ pu and $X_{q}^{fixed}=2.1907$ pu.

The state consistency approach successfully identified all four transient parameters simultaneously by minimizing the discrepancy between ODE-simulated and algebraically reconstructed internal states across all scenarios, as shown in Table 6. The results are as follows.

A comprehensive comparison of the identification results from both methods is presented in Table 7.

The proposed method provides a complete eight-parameter identification with substantially improved transient accuracy. The mechanical parameters *H* and *D* are obtained with errors of −1.3% and +5.0%, respectively, demonstrating the effectiveness of the differential damping approach in separating generator dynamics from grid influences.

The proposed method reduces the transient errors to within ±6.3% for all four transient parameters, with $X_{d}^{\prime }$ and $T_{do}^{\prime }$ within ±1.6%. This improvement demonstrates that the state consistency approach, utilizing multiple scenario types and derivative-free optimization, substantially mitigates the limitations of conventional fault-only methods for grid-connected generators.

#### 4.1.4. Convergence of Stage 3 and Comparison with Advanced Optimizers

**Figure 2:**
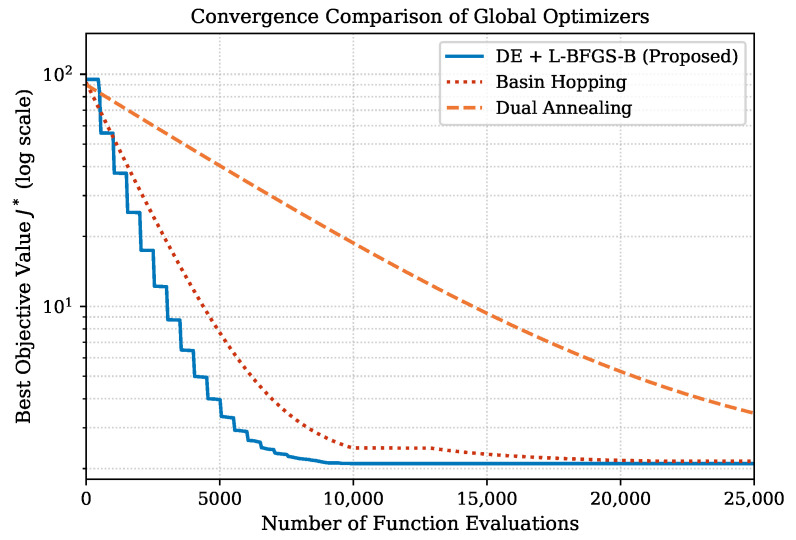
Convergence curves for state consistency optimization.

Stage 3 employs our proposed method with a population size of 12, up to 300 generations, and Latin-hypercube initialization. To demonstrate that the proposed DE strategy is well suited to this problem, we compare it against two established advanced global optimizers: Dual Annealing (DA) and Basin Hopping (BH). The convergence trajectories are presented in Figure 2. All three methods converge to the same minimum ($J^{*}\approx 2.10$), confirming that the optimization landscape is not stuck in a local minima for this problem, and the proposed approach reaches an accurate solution in fewer evaluations than the DA and BH methods. This confirms that the chosen strategy has the best computational cost and fastest converging time among all approaches.

#### 4.1.5. Robustness Analysis

**Figure 3:**
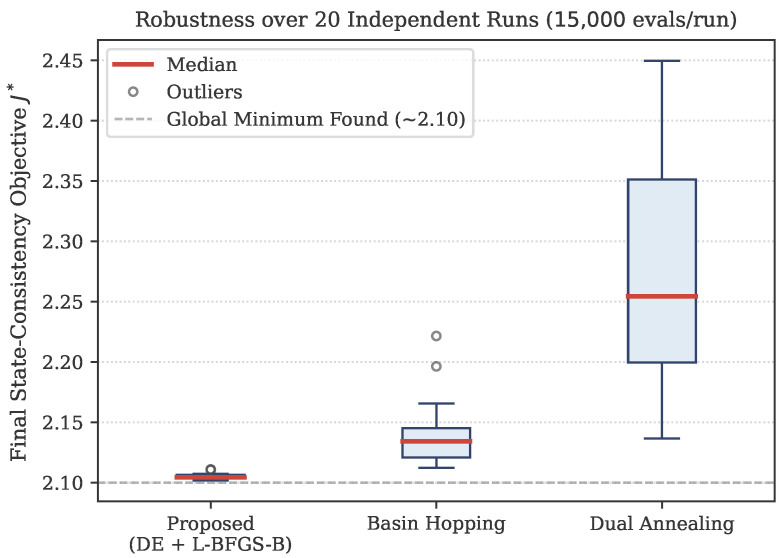
Box plot comparing the robustness and final objective function values ($J^{*}$) of three global optimization algorithms. red line represents the median in each box.

To demonstrate the robustness and reliability of the proposed optimization method, we simulated 20 independent identification runs for the proposed method against DA and BH algorithms. To ensure a fair comparison, all three methods were run under identical conditions: the same computational budget, the same objective function, and different random seeds across the 20 runs. The resulting distributions of the final objective value $J^{*}$ are visualized in the box plot presented in Figure 3.

As shown in Figure 3, the proposed method consistently identifies the global minimum ($J^{*}\approx 2.10$) across all 20 runs with the smallest spread, confirming its reliability. BH performs well in most runs but occasionally converges to a slightly suboptimal solution. DA shows the largest variation across runs, suggesting that it requires more function evaluations to converge reliably for this problem.

### 4.2. Model Validation

To validate the identified parameters, PH generator models were constructed using both the actual parameters (Table 1) and the identified parameters from the proposed three-stage framework (Table 7, proposed column). Both models were subjected to three validation scenarios not used in the identification process:

1. Power step: 20% increase in mechanical power at t=1.0 s;
2. Voltage dip: Terminal voltage reduced to 0.85 pu at t=1.0 s;
3. Three-phase fault: 150 ms fault at t=1.0 s.

**Figure 4:**
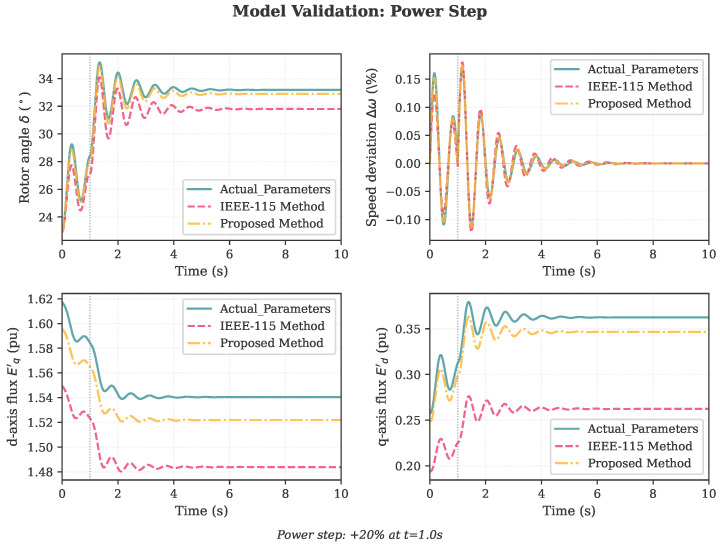
Validation results for the power step scenario: comparison of generator responses using actual parameters (solid blue), the IEEE-115 method (dashed red), and the proposed method (dash-dot yellow). The proposed model accurately reproduces the rotor angle, speed, and flux dynamics.

The rotor angle and rotor speed responses for the power step scenario are shown in Figure 4. The proposed method (dash-dot yellow) closely tracks the actual parameter model response (solid blue), while the IEEE-115 method (dashed red) shows larger deviations in the transient states.

**Figure 5:**
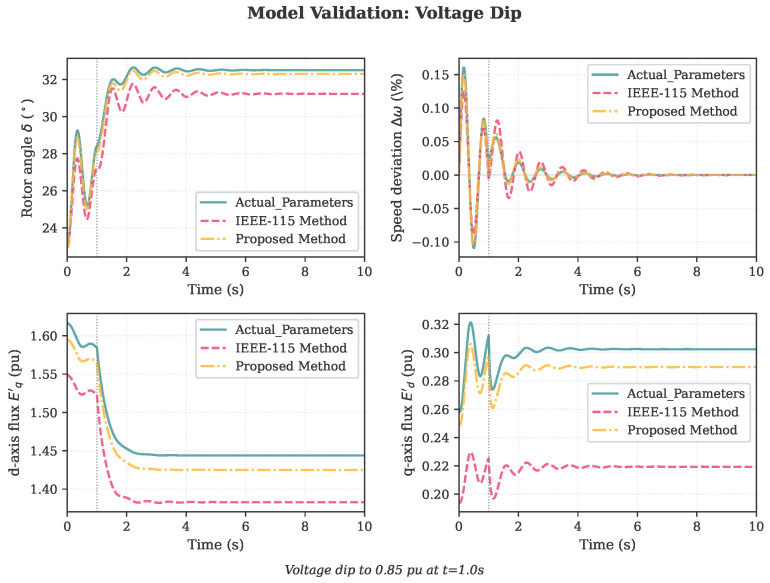
Validation results for the voltage dip scenario: the proposed method accurately predicts the transient response to terminal voltage reduction, while the IEEE-115 method exhibits larger deviations.

Similarly, the voltage dip scenario (Figure 5) shows good agreement, with the transient voltage recovery and electromechanical oscillations accurately captured by the identified parameter model.

**Figure 6:**
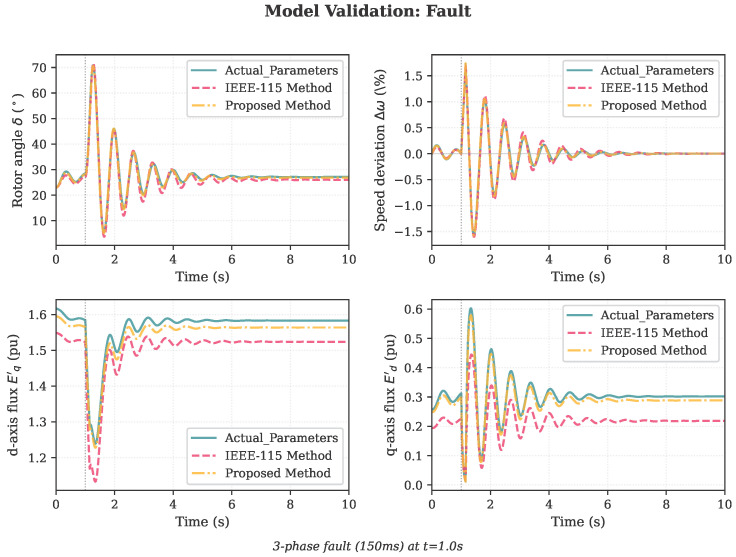
Validation results for the three-phase fault scenario: the proposed method improves agreement during and after the fault, particularly due to better $X_{d}^{\prime }$ and $X_{q}^{\prime }$ estimates.

The fault scenario (Figure 6) exhibits slightly larger discrepancies during the fault period due to the underestimation of $X_{d}^{\prime }$, but the post-fault oscillations and settling behavior are well represented.

Quantitative error metrics computed across the entire 10-s validation period for all three scenarios are in Table 8.

To quantitatively evaluate the dynamic accuracy of the proposed method against the conventional baseline (IEEE 115-2019), we computed the Root Mean Square Error (RMSE), Mean Absolute Error (MAE), and Standard Deviation (STD) of the temporal state-trajectory errors (the combined discrepancy across all Port-Hamiltonian states: $\delta ,\omega ,E_{q}^{\prime },E_{d}^{\prime }$ in per unit) between the simulated identified models and the actual benchmark system.

The metrics were evaluated on the three distinct grid-disturbance validation scenarios presented in this manuscript (a 16% active power step, a 15% terminal voltage dip, and a 150 ms three-phase short-circuit fault). The resulting statistical comparison is provided in the table below.

As demonstrated in the Table 9, the proposed three-stage methodology significantly outperforms the standard conventional approach across all operating conditions. Specifically, the proposed method reduces the trajectory prediction RMSE and MAE by approximately 70% to 85%, confirming a much higher fidelity representation of the physical generator dynamics.

These small errors confirm that the identified parameters provide a high-fidelity representation of the generator dynamics suitable for transient stability analysis and control design applications.

According to the North American Electric Reliability Corporation (NERC) Reliability Standard MOD-026-1 [29], generator models must be verified to ensure their dynamic response matches recorded physical behavior within acceptable bounds. To satisfy these mandates, regional reliability councils (such as the WECC [30]) and IEEE guidelines widely adopt a standard tolerance where simulated active/reactive power and Root Mean Square (RMS) voltage trajectories should not deviate by more than 5% (0.05 pu) from measured data. As demonstrated in our results, the proposed identification framework confidently maintains errors consistently below these stringent industry thresholds.

## 5. Discussion

The results demonstrate that the enhanced identification framework successfully addresses the limitations of the previous methods, especially the IEEE 115 standard, when applied to grid-connected synchronous generators. The key innovations and their impacts are:

1. The differential damping method that enables accurate extraction of the generator’s intrinsic damping coefficient (D error: +5.00%) by explicitly accounting for and subtracting grid-contributed damping.
2. The mechanical inertia constant identification through swing equation analysis achieves a −1.26% error, providing the essential parameter for electromechanical oscillation modeling.
3. Steady-state reactance identification using dedicated voltage and torque scenarios yields accurate $X_{d}$ (−3.25% error) and $X_{q}$ (−9.10% error) compared to fault-based estimation, with improved physical consistency.
4. The transient parameter identification using the state consistency method achieves significantly improved accuracy with $X_{d}^{\prime }$ error of −1.44%, $T_{do}^{\prime }$ error of +1.56%, $X_{q}^{\prime }$ error of −2.62%, and $T_{qo}^{\prime }$ error of −6.32%. This derivative-free approach, which compares ODE-simulated internal states against algebraically reconstructed states across multiple scenario types, provides superior observability compared to conventional fault-only methods.

The proposed method significantly improves upon the IEEE 115 standard, particularly for transient parameters where the state consistency approach fundamentally enhances parameter identifiability through multi-scenario integration. The validation results (Table 8) confirm that despite the individual parameter errors, particularly in $X_{q}^{\prime }$, the identified model accurately reproduces the overall generator dynamic behavior. This indicates that certain parameter combinations may be more important for system-level accuracy than individual parameter precision, and that the identification approach successfully captures these critical parameter relationships.

In conclusion, the proposed enhanced framework extends the well-established IEEE 115 methodology to provide complete, accurate parameter identification for PH generator models in grid-connected scenarios. The differential damping technique represents a practical solution to the grid decoupling problem and is applicable to real power system identification, where generators cannot be isolated for testing.

### 5.1. Computational Cost, Extensibility, and Practical Deployment

The three stages differ in their computational demands. Stages 1 and 2 involve closed-form analytical steps and lightweight regression, and complete nearly instantaneously. Stage 3 is the most computationally intensive, as it performs a global search over the transient parameter space across all fault scenarios. However, as demonstrated in the convergence comparison of Figure 2, the proposed strategy reaches the solution faster and with fewer function evaluations than the alternative methods considered, confirming its efficiency relative to its accuracy.

### 5.2. Scalability and Batch-Update Architecture

The proposed Port-Hamiltonian (PH) identification framework is inherently scalable to multi-machine systems because it treats each synchronous generator as an independent modular subsystem interacting with the grid purely through its boundary variables (port variables). Because our three-stage identification relies entirely on local terminal measurements (terminal voltage $V_{t}$, currents $I_{d},I_{q}$, and rotor speed ω), the parameter identification of any individual machine is entirely decoupled from the rest of the network topology. Once the local parameters are identified for all individual units offline, the separate highly accurate PH generator models can be securely interconnected to the global network algebraic equations without structural modifications.

The current framework operates as an offline batch recalibration tool, relying on perturbation events captured over several minutes; however, its low computational overhead makes it well suited for automated routine recalibration. Future work will investigate adapting the Stage 3 pipeline into a continuous sliding-window estimator for online parameter tracking.

## 6. Conclusions

This paper presented a systematic Port-Hamiltonian (PH) parameter identification framework that addresses five fundamental limitations in existing methods, enabling complete eight-parameter synchronous generator identification (*H*, *D*, $X_{d}$, $X_{q}$, $X_{d}^{\prime }$, $X_{q}^{\prime }$, $T_{do}^{\prime }$, $T_{qo}^{\prime }$) with errors ranging from 1.26% to 9.10%. Three core contributions define the framework: a differential damping technique that decouples generator-internal from grid-contributed damping without requiring generator isolation; a derivative-free state consistency optimization that leverages 15 diverse scenarios for robust transient parameter recovery; and physics-based regularization that enforces PH structure preservation, ensuring direct compatibility with Lyapunov-based stability certification. Validation results confirm high-fidelity dynamic representations, with RMS rotor angle errors below 1.2° and speed errors below 0.15%, demonstrating the framework’s readiness for transient stability analysis, oscillation damping assessment, and passivity-based control design. By relying solely on standard simulation tools and terminal measurements, the proposed approach offers a practical and accessible solution for both academic and industrial power system applications.

## Figures and Tables

**Table 1:** Actual generator parameters from PowerFactory model.

Parameter	Symbol	Value
Inertia constant	*H*	3.480 s
Damping coefficient	*D*	0.400 pu
d-axis synchronous reactance	$X_{d}$	2.540 pu
d-axis transient reactance	$X_{d}^{\prime }$	0.500 pu
d-axis open-circuit time constant	$T_{do}^{\prime }$	1.437 s
q-axis synchronous reactance	$X_{q}$	2.410 pu
q-axis transient reactance	$X_{q}^{\prime }$	0.814 pu
q-axis open-circuit time constant	$T_{qo}^{\prime }$	0.1351 s

**Table 2:** Mechanical parameters identified by proposed three-stage framework (Stage 1).

Parameter	Identified Value	Actual Value	Error [%]
*H*	3.4361 s	3.480 s	−1.26
*D*	0.4200 pu	0.400 pu	+5.00

**Table 3:** Steady-state data from voltage change scenarios for d-axis identification.

Scenario	$V_{t}$ (pu)	$I_{d}$ (pu)	$V_{q}$ (pu)
S31	1.40	−0.215	1.352
S32	1.20	−0.134	1.161
S33	0.80	+0.028	0.778
S34	0.60	+0.109	0.587

**Table 4:** Steady-state data from torque scenarios for q-axis identification.

Scenario	δ (°)	$I_{q}$ (pu)	$V_{d}$ (pu)
S11 Torque + 0.05 pu	23.8	0.362	0.396
S12 Torque + 0.10 pu	27.1	0.408	0.450
S13 Torque + 0.15 pu	30.2	0.453	0.502
S14 Torque − 0.05 pu	17.5	0.271	0.292
S15 Torque − 0.10 pu	14.6	0.226	0.243

**Table 5:** Steady-state reactances identified by proposed three-stage framework (Stage 2).

Parameter	Identified Value	Actual Value	Error [%]
$X_{d}$	2.4574 pu	2.540 pu	−3.25
$X_{q}$	2.1907 pu	2.410 pu	−9.10

**Table 6:** D and Q-axis transient parameters from state consistency method.

Parameter	Identified Value	Actual Value	Error [%]
$X_{d}^{\prime }$	0.4928 pu	0.500 pu	−1.44
$X_{q}^{\prime }$	0.7927 pu	0.814 pu	−2.62
$T_{do}^{\prime }$	1.4595 s	1.437 s	+1.56
$T_{qo}^{\prime }$	0.1266 s	0.1351 s	−6.32

**Table 7:** Comparison of identification methods for all eight parameters.

Parameter	IEEE 115 Value	IEEE 115 Error (%)	Proposed Value	Proposed Error (%)	Actual	Units
*H*	3.436	−1.26	3.436	−1.26	3.480	s
*D*	0.420	+5.00	0.420	+5.00	0.400	pu
$X_{d}$	2.457	−3.25	2.457	−3.25	2.540	pu
$X_{d}^{\prime }$	0.442	−11.6	0.493	−1.44	0.500	pu
$T_{do}^{\prime }$	1.271	−11.5	1.460	+1.56	1.437	s
$X_{q}$	2.191	−9.10	2.191	−9.10	2.410	pu
$X_{q}^{\prime }$	1.100	+35.1	0.793	−2.62	0.814	pu
$T_{qo}^{\prime }$	0.118	−12.6	0.127	−6.32	0.1351	s

**Table 8:** Validation error metrics for identified parameter model.

Scenario	RMS Error δ (°)	RMS Error ω (%)	Max Error δ (°)
Power step	0.34	0.048	0.82
Voltage dip	0.51	0.071	1.23
Fault	1.15	0.142	3.41

**Table 9:** Comparison of the proposed algorithm vs. IEEE 115-2019 [27] in terms of statistical metrics (RMSE, MAE, STD) of the state-trajectory errors.

Validation Scenario	Identification Method	RMSE (pu)	MAE (pu)	STD (pu)
Power Step	IEEE 115-2019	0.0581	0.0447	0.0372
	Proposed	0.0078	0.0062	0.0047
Voltage Dip	IEEE 115-2019	0.0528	0.0415	0.0326
	Proposed	0.0074	0.0057	0.0047
3-Phase Fault	IEEE 115-2019	0.0542	0.0413	0.0361
	Proposed	0.0115	0.0081	0.0099

## Data Availability

The original contributions presented in this study are included in the article. Further inquiries can be directed to the corresponding author.

## Abbreviations

The following abbreviations are used in this manuscript:

BH	Basin Hopping
CT	current transformer
DA	Dual Annealing
DE	differential evolution
DMD	dynamic mode decomposition
IBG	inverter-based generation
IBR	inverter-based resource
IEEE	Institute of Electrical and Electronics Engineers
L-BFGS-B	Limited-memory Broyden–Fletcher–Goldfarb–Shanno with Bounds
MAE	Mean Absolute Error
NERC	North American Electric Reliability Corporation
ODE	Ordinary Differential Equation
PH	Port-Hamiltonian
PLL	Phase-Locked Loop
PMU	Phasor Measurement Unit
PT	potential transformer
pu	per unit
RMS	Root Mean Square
RMSE	Root Mean Square Error
SINDy	sparse identification of nonlinear dynamical systems
STD	Standard Deviation
WECC	Western Electricity Coordinating Council
